# TNF signaling mediates an enzalutamide-induced metastatic phenotype of prostate cancer and microenvironment cell co-cultures

**DOI:** 10.18632/oncotarget.4535

**Published:** 2015-07-30

**Authors:** Kai Sha, Shuyuan Yeh, Chawnshang Chang, Kent L. Nastiuk, John J. Krolewski

**Affiliations:** ^1^ Department of Pathology and Laboratory Medicine, University of Rochester, School of Medicine and Dentistry; Rochester, NY 14642, USA; ^2^ Department of Urology, University of Rochester, School of Medicine and Dentistry; Rochester, NY 14642, USA; ^3^ Department of Radiation Oncology, University of Rochester, School of Medicine and Dentistry; Rochester, NY 14642, USA; ^4^ Wilmot Cancer Institute, University of Rochester, School of Medicine and Dentistry; Rochester, NY 14642, USA; ^5^ Department of Cancer Genetics, Roswell Park Cancer Institute, Buffalo, NY 14263; ^6^ Center for Personalized Medicine, Roswell Park Cancer Institute, Buffalo, NY 14263

**Keywords:** CCL2, enzalutamide, metastasis, microenvironment, TNF

## Abstract

The dramatic responses tumors display to targeted therapies are limited by acquired or pre-existing mechanisms of therapy resistance. We recently discovered that androgen receptor blockade by the anti-androgen enzalutamide paradoxically enhanced metastasis and that these pro-metastatic effects were mediated by the chemoattractant CCL2. CCL2 is regulated by TNF, which is negatively regulated by androgen signaling. Thus, we asked if TNF mediates the pro-metastatic effects of enzalutamide. We found that androgen withdrawal or enzalutamide induced *TNF* mRNA and protein secretion in castration resistant prostate cancer (C4-2) cells, but not in macrophage-like (THP1) or myofibroblast-like (WPMY1) cells. Androgen deprivation therapy (ADT) induced autocrine CCL2 expression in C4-2 (as well as a murine CRPC cell line), while exogenous TNF induced CCL2 in THP1 and WPMY1. TNF was most potent in myofibroblast cultures, suggesting ADT induces CCL2 via paracrine interactions within the tumor microenvironment. A soluble TNF receptor (etanercept) blocked enzalutamide-induced CCL2 protein secretion and mRNA, implying dependence on secreted TNF. A small molecule inhibitor of CCR2 (the CCL2 receptor) significantly reduced TNF induced migration, while etanercept inhibited enzalutamide-induced migration and invasion of C4-2. Analysis of human prostate cancers suggests that a TNF-CCL2 paracrine loop is induced in response to ADT and might account for some forms of prostate cancer therapy resistance.

## INTRODUCTION

The concept of ‘oncogene addiction’ [1] has given rise to targeted cancer therapy, which is effective in treating cancers dependent on the corresponding oncogenic driver [2]. Examples include the epidermal growth factor receptor (EGFR) and the androgen receptor (AR). However, the dramatic responses to targeted therapy are limited by pre-existing or acquired therapy resistance [3]. The best understood mechanisms are secondary missense mutations in driver genes. The T790M ‘gateway’ mutation in the EGFR produces erlotinib and gefitinib resistance [4–6], while the F876L mutation in the AR converts enzalutamide from an antagonist to an agonist [7–9]. Epigenetic mechanisms can alter the expression of splice variants, such as those encoding ligand-independent versions of the AR, which are expressed at increased levels in treatment resistant prostate cancers [10]. Non-genetic mechanisms are not as well understood, but may play a role in treatment failure. In this report, we show that androgen deprivation therapy can paradoxically activate a pro-tumorigenic signaling mechanism that is dependent on paracrine interactions within the tumor microenvironment.

We (SY and CC) recently discovered that androgen deprivation therapy (ADT) unexpectedly enhances the metastatic phenotype of castration resistant prostate cancer (CRPC), even as these therapies inhibit tumor growth [11–13]. Specifically, treatment with the anti-androgens bicalutamide and enzalutamide inhibited proliferation but induced macrophage migration and tumor cell invasion *in vitro* as well as distant metastases in orthotopic tumor models of CRPC [11]. This may reflect a divergence between the effects of AR signaling on proliferation compared to the effects on the metastatic phenotype. These pro-metastatic effects are mediated by CCL2 [11, 12], a chemokine also known as monocyte chemoattractant protein-1 that binds the cognate receptor CCR2 to induce chemotaxis [14, 15]. Monocytes are a major source of CCL2 [15], but CCL2 is also produced by a variety of cells in tumors, including epithelial tumor cells and the cellular components of the tumor microenvironment, such as endothelium, stroma and tumor-associated macrophages (TAMs) [14]. In addition, studies of prostate cancer (PCa) patient serum and/or tumor tissue samples support a role for CCL2 in ADT-induced metastasis [11, 12, 16]. We (KLN and JJK) have also previously demonstrated that TNF is negatively regulated by androgens [17]. Specifically, castration induces *TNF* mRNA in rodent prostatic stroma. Promoter analysis has shown that CCL2 is regulated by TNF via NFκB [18]. Indeed, it has been reported that TNF induces CCL2 expression in ovarian cancer cells [19] as well as sensory neurons [20–22], and vascular smooth muscle cells [23]. Given these two sets of previous findings from our laboratories, we tested the hypothesis that TNF signaling is required for enzalutamide induced metastasis of CRPC via CCL2.

## RESULTS

### Androgen deprivation induces TNF expression

To address the role of TNF in metastasis following androgen deprivation, we initially employed three cell lines, representing CRPC (C4-2), prostate stromal myofibroblasts (WPMY-1) and tumor associated macrophages (THP-1), either alone, in co-culture or via conditioned media, to simulate the *in vivo* context of PCa. C4-2, a sub-line of the human androgen-dependent LNCaP prostate cancer cell line, derived by selecting for growth as a xenograft in a castrated athymic nude mouse [24–26], is a well-established cell line model for CRPC. WPMY-1 is an SV40 large-T antigen-immortalized myofibroblast cell line (expressing smooth muscle α-actin and vimentin), derived from a cancerous human prostate [26]. THP-1 [27] is derived from a human acute monocytic leukemia, displays monocytic markers, has phagocytic activity and expresses CCR2 [28], indicating that it is a model for TAMs [29].

**Figure 1:**
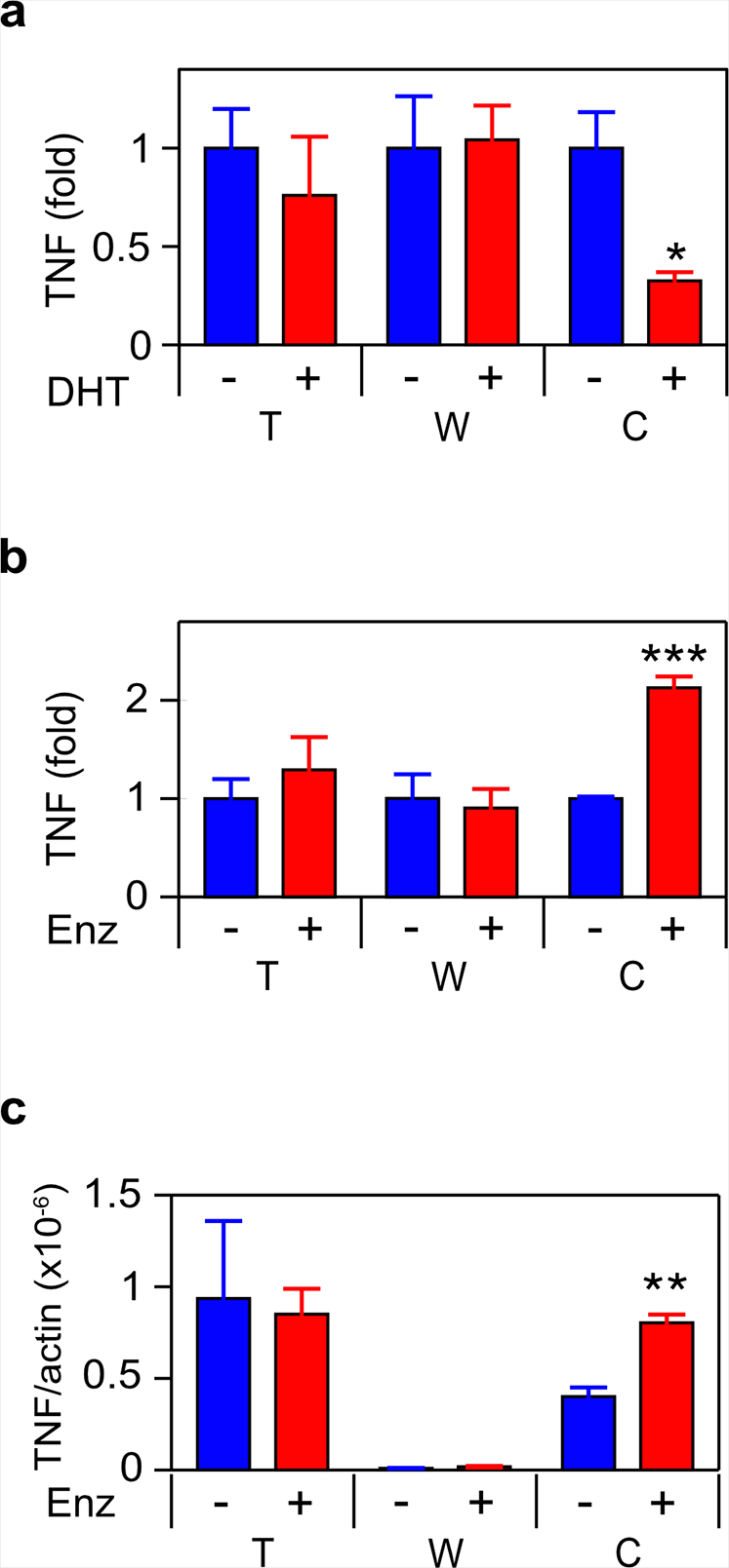
ADT induces TNF expression in CRPC **a. b.** THP1 (T), WPMY-1 (W) or C4-2 (C) cells were treated for 72 h with vehicle (−) or 10 nM DHT (+) (a) or 10 μM enzalutamide (Enz; +) (b) TNF was assayed by ELISA (*n* ≥ 3). **c.** mRNA from cultures identical to (b) was quantitated by RT-PCR. TNF mRNA levels, normalized to Δ-actin, are shown (*n* = 3). Treated (red) and untreated (blue) cultures compared by Student's unpaired *t*-test. *p < 0.05, ***p < 0.001.

**Figure 2:**
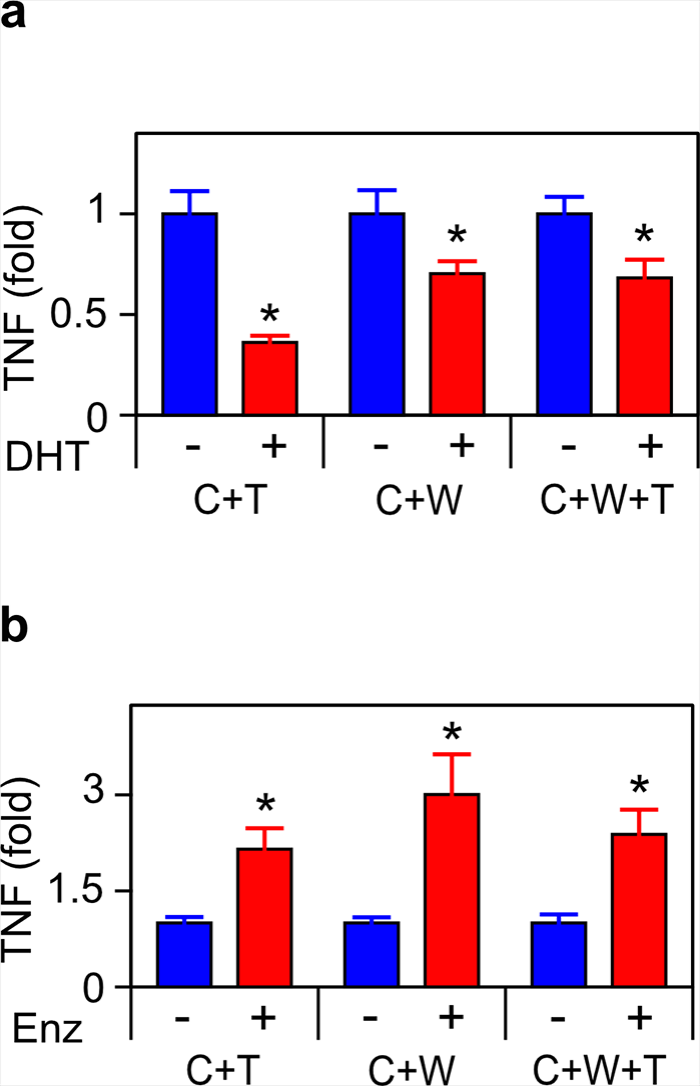
ADT induces TNF secretion in tumor/microenvironment co-cultures Co-cultures, as indicated in Figure 1, were treated for 72 h with vehicle (−) or 10 nM DHT (+) **a.** or 10 μM enzalutamide (Enz) (+) **b.** TNF was assayed by ELISA (*n* = 3). Treated (red) and untreated (blue) cultures compared by Student's unpaired *t*-test. *p < 0.05.

Following treatment with dihydrotestosterone (DHT), TNF secretion was reduced in C4-2 (Figure 1a). Conversely, treatment of C4-2 with the anti-androgen enzalutamide induced TNF secretion and an increase in mRNA expression (Figure 1b–1c). The coordinate increase in both protein and mRNA is consistent with transcriptional repression of the *TNF* gene by the AR. However, we cannot exclude effects on mRNA stability. Neither DHT nor enzalutamide affected TNF expression in the TAM-like THP-1 or stromal myofibroblast-like WPMY-1 cell lines. Similarly, in the rat stroma-derived PS-1 cells, there was no change in TNF expression in response to enzalutamide (data not shown). The anti-androgen bicalutamide and the synthetic androgen R1881 had analogous effects to enzalutamide and DHT, respectively (Supplementary Figure S1). Similar levels of TNF secretion, following DHT withdrawal or enzalutamide treatment, were observed when C4-2 cells were co-cultured with THP-1 and/or WPMY-1 cells (Figure 2).

### TNF is required for CCL2 secretion

**Figure 3:**
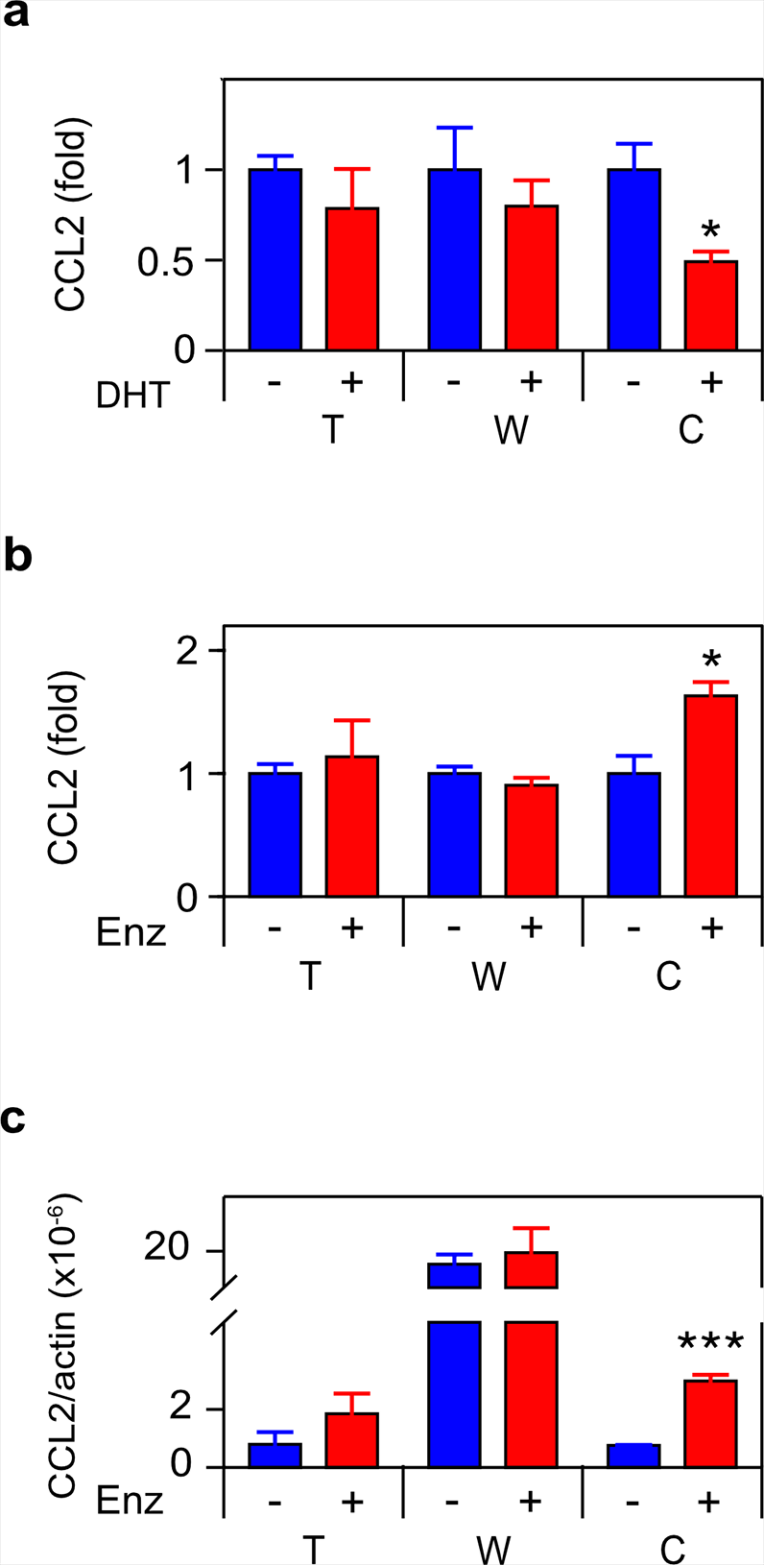
ADT induces CCL2 expression in CRPC cells **a. b.** Cells as indicated in Figure 1 were treated for 72 h with vehicle (−) or 10 nM DHT (+) (a) or 10 μM enzalutamide (Enz) (+) (b) CCL2 was assayed by ELISA (*n* ≥ 3). **c.** mRNA from cultures identical to (b) were quantitated by RT-PCR. CCL2 mRNA levels, normalized to Δ-actin, are shown (*n* = 3). Treated (red) and untreated (blue) cultures compared by Student's unpaired *t*-test. *p < 0.05, **p < 0.01.

**Figure 4:**
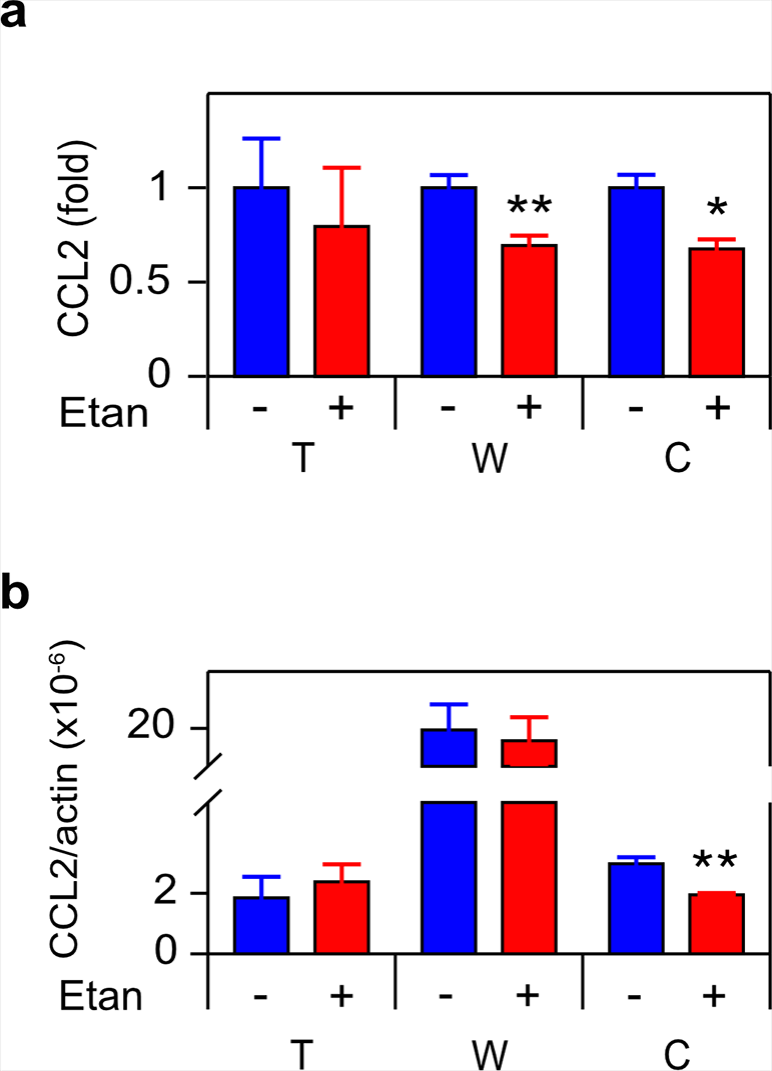
TNF is required for enzalutamide-induced CCL2 expression in human CRPC **a.** Cells indicated as in Figure 1 were cultured in the presence of 10 μM enzalutamide and either vehicle (−) or 1 μg/mL etanercept (Etan) (+), and assayed for CCL2 by ELISA 72 h later (*n* ≥ 3). **b.** mRNAs from cultures identical to **a.** were quantitated by RT-PCR. *CCL2* mRNA levels, normalized to Δ-actin, are shown (*n* = 3). Treated (red) and untreated (blue) cultures compared by Student's unpaired *t*-test. *p < 0.05, **p < 0.01.

Since we previously observed that androgen deprivation induced CCL2 [11], we measured CCL2 secretion and mRNA in the cultures examined in Figure 1 and observed an identical pattern, correlating TNF and CCL2 expression at the protein and mRNA levels (Figure 3). CCL2 and TNF secretion were also coincident in C4-2 treated with bicalutamide or R1881 (Supplementary Figure S2). To determine if CCL2 expression was regulated by TNF, we employed etanercept, a soluble receptor composed of the extracellular domain of the p75 TNF receptor (TNFR2) fused to the Fc portion of the immunoglobulin protein. Etanercept binds TNF avidly and with high specificity [30], making this an ideal reagent for testing the role of secreted or membrane bound TNF in mediating intracellular signaling events. Figure 4 demonstrates that etanercept blocked enzalutamide induced secretion of the CCL2 protein (Figure 4a) as well as the induction of *CCL2* mRNA levels (Figure 4b). This suggests autocrine TNF signaling can regulate transcription of the *CCL2* gene (or, alternatively, *CCL2* mRNA stability) in C4-2 cells. Etanercept also blocked CCL2 secretion, but not the mRNA level, in WPMY-1 myofibroblasts (Figure 4). However, enzalutamide did not induce TNF production at either the protein or mRNA level in WPMY-1 (Figure 1b–1c).

**Figure 5:**
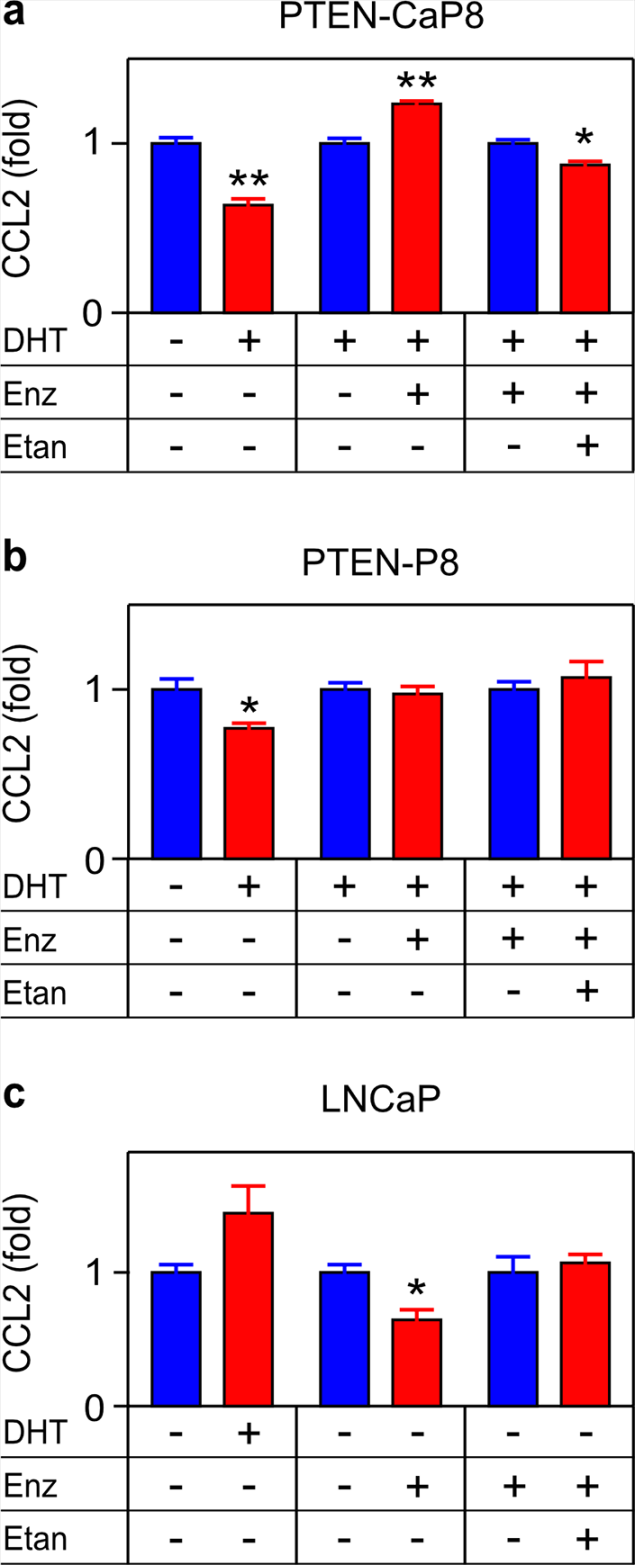
TNF is required for enzalutamide-induced CCL2 expression in a murine CRPC cell line PTEN-CaP8 **a.** PTEN-P8 **b.** and LNCaP **c.** were treated with vehicle (−), 10 nM DHT (DHT, +), 10 μM enzalutamide (Enz, +) or 1 μg/mL etanercept (Etan, +) as indicated. Secreted CCL2 was measured by ELISA after 72 h (*n* ≥ 3). Treated (red) and untreated (blue) cultures compared by Student's unpaired *t*-test. *p < 0.05, **p < 0.01.

Figures 1–4 indicate that TNF signaling mediates ADT induced transcription and secretion of CCL2 by C4-2 CRPC cells. To determine if our observations in C4-2 cells are representative of the response of CRPC to ADT, we examined PTEN-CaP8, a murine CRPC cell line derived from prostate-specific *Pten* null mice homozygously deleted for PTEN [31]. To do this, we replicated the experiments shown in Figures 3a–3b and Figure 4a, employing PTEN-CaP8 in place of C4-2 cells and found that PTEN-CaP8 cells responded the same as C4-2 cells (Figure 5a). Specifically, DHT reduced, and enzalutamide induced, CCL2 secretion in PTEN-CaP8 cells and etanercept blocked enzalutamide-induced CCL2 production (Figure 5a). Thus, TNF-mediated CCL2 induction may be a typical response of CRPC to ADT. To verify that this response is a feature of CRPC cell lines, we examined the isogenic parental lines that are the progenitors of PTEN-CaP8 and C4-2. These cell lines (PTEN-P8 and LNCaP, respectively) resemble localized prostate cancers; that is, these are less advanced versions of the corresponding CRPC cell line. For both PTEN-P8 and LNCaP, the effects of blocking androgen signaling were less pronounced. Importantly, even when regulated by ADT, etanercept did not block CCL2 production (Figure 5b, 5c), indicating that these less advanced cell lines are distinct from the CRPC cell lines. Taken together, this suggests that ADT-induced, TNF-dependent CCL2 production is a feature of CRPC.

### TNF blockade reduces paracrine CCL2 secretion

**Figure 6:**
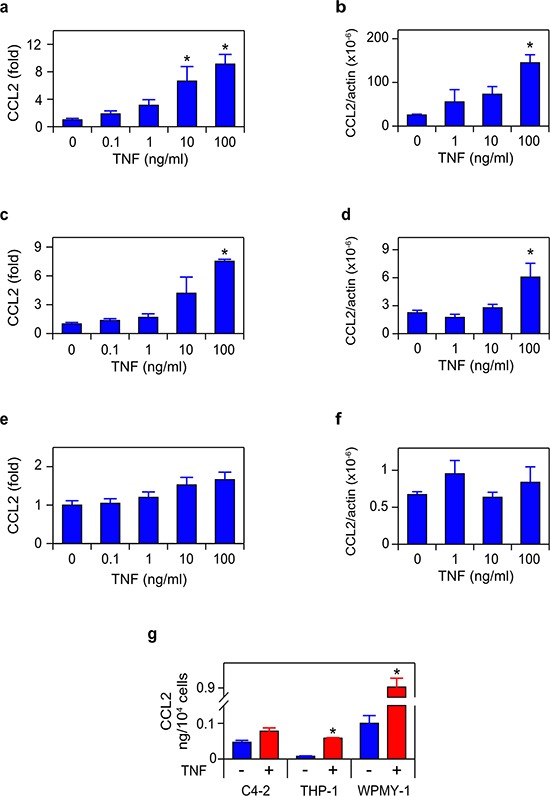
TNF induces CCL2 in microenvironment-derived cells **a. c. e.** WPMY-1 (a) THP1 (c) and C4-2 (e) were treated with TNF for 72 h, as indicated. Secreted CCL2 was measured by ELISA (*n* = 3). **b. d. f.** RNA was extracted from WPMY-1 (b) THP-1 (d) and C4-2 (f) treated as in (a, c, e) CCL2 mRNA levels, normalized to Δ-actin, are shown (*n* = 3). **g.** The amount of CCL2 secreted by the indicated cell lines, following 72 h treatment with vehicle (red) or 100 ng/mL TNF (blue). Data re-plotted from (a, c, e) Differences were assessed by one-way ANOVA, followed by Tukey-Kramer HSD test (a–f). *p < 0.05.

To directly test the requirement for TNF, C4-2, THP-1 and WPMY-1 cells were treated with TNF, and CCL2 secretion and mRNA was measured (Figure 6). The two microenvironment-derived cell lines (THP-1 and WPMY-1, representing TAMs and myofibroblast stromal cells, respectively) demonstrated increased CCL2 expression at both the protein and mRNA levels, in response to TNF (Figure 6a–6d).

**Figure 7:**
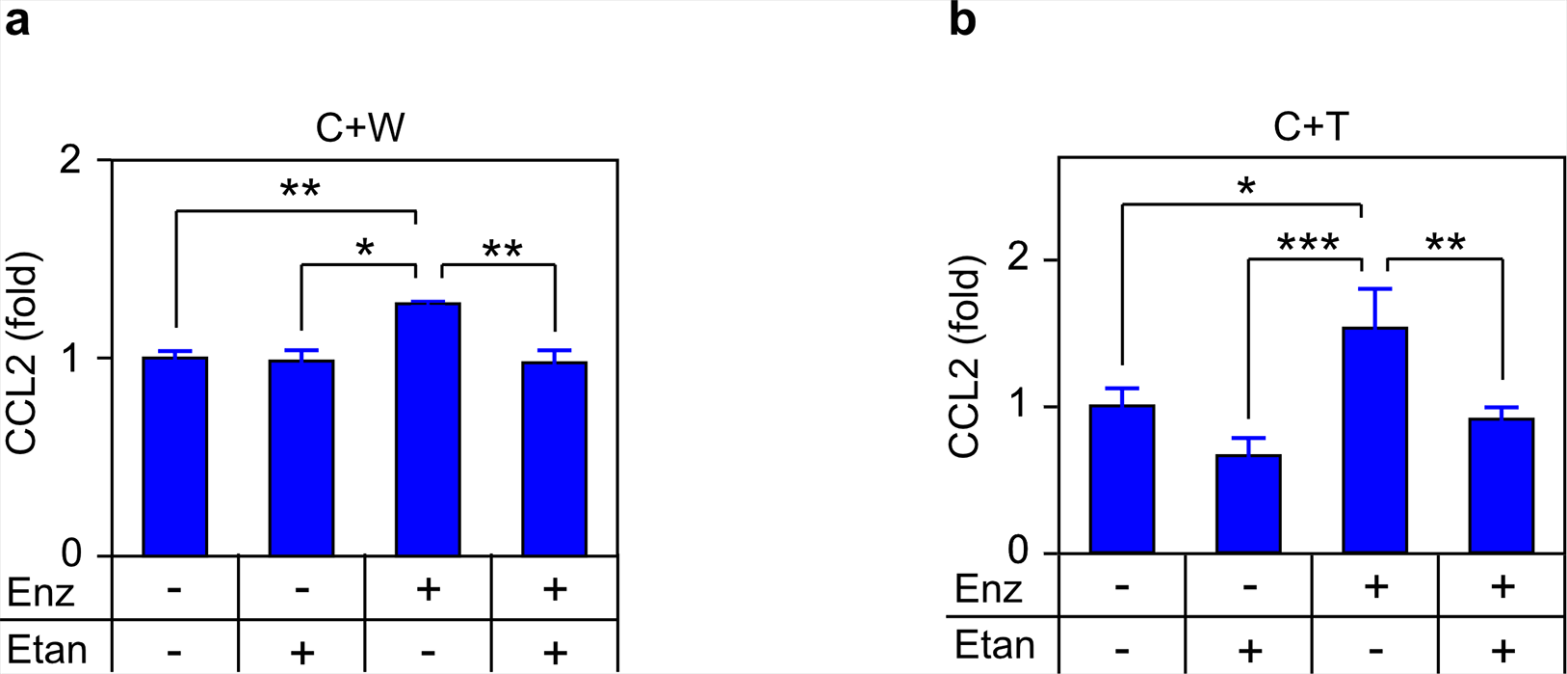
TNF is required for enzalutamide-induced CCL2 secretion in co-cultures Mixed cultures of C4-2 plus WPMY-1 (C+W;) **a.** or C4-2 PCa plus THP1 (C+T;) **b.** were treated with vehicle (−), 10 μM enzalutamide (Enz, +) or 1 μg/mL etanercept (Etan, +) for 72 h, as indicated. Secreted CCL2 was measured by ELISA (*n* ≥ 3). Differences were assessed by one-way ANOVA followed by Tukey-Kramer HSD test. *p < 0.05, **p < 0.01, ***p < 0.001.

Surprisingly, C4-2 cells, which produce CCL2 in a TNF-dependent, autocrine manner (Figures 3–4), did not show significant modulation of CCL2 in response to TNF (Figure 6e–6f), even though exogenous TNF was employed at doses that exceed the concentration of soluble TNF detected in C4-2 media following androgen deprivation (5–10 pg/mL; data not shown). This suggests that TNF concentrations determined by ELISA in cell culture media might not reflect the concentration available to receptors on the C4-2 cell surface. Indeed, TNF is expressed in a membrane-bound form, which is cleaved by the extracellular protease TACE to generate the soluble (‘secreted’) form of the cytokine [32]. Since membrane-bound TNF is biologically active and cannot be detected in ELISA assays of cell culture media, we may have significantly underestimated the biologically effective TNF concentration that is required to trigger the autocrine effects in the single cell culture conditions we employed in Figures 3 and 4. This suggests the possibility that microenvironment cell lines, which are more responsive to TNF (see Figure 6a–6d versus Figure 6e–6f), might be the relevant source of CCL2 in the *in vivo* tumor context. Thus, the production of CCL2 may be mediated by paracrine interactions between the tumor cells and the microenvironment, rather than autocrine production of CCL2 by the C4-2 prostate cancer tumor cells. To test this, we mixed C4-2 tumor cells with either THP-1 or WPMY-1 cells, and blocked androgen binding and signaling in these co-cultures, using the androgen antagonist enzalutamide. In both mixed cultures (C4-2 tumor cells plus WPMY-1 myofibroblasts (Figure 7a), and C4-2 tumor cells plus THP-1 TAM-like cells (Figure 7b)), enzalutamide induced CCL2 and etanercept blocked CCL2 induction, consistent with our proposed paracrine mechanism.

### TNF is required for migration and invasion

**Figure 8:**
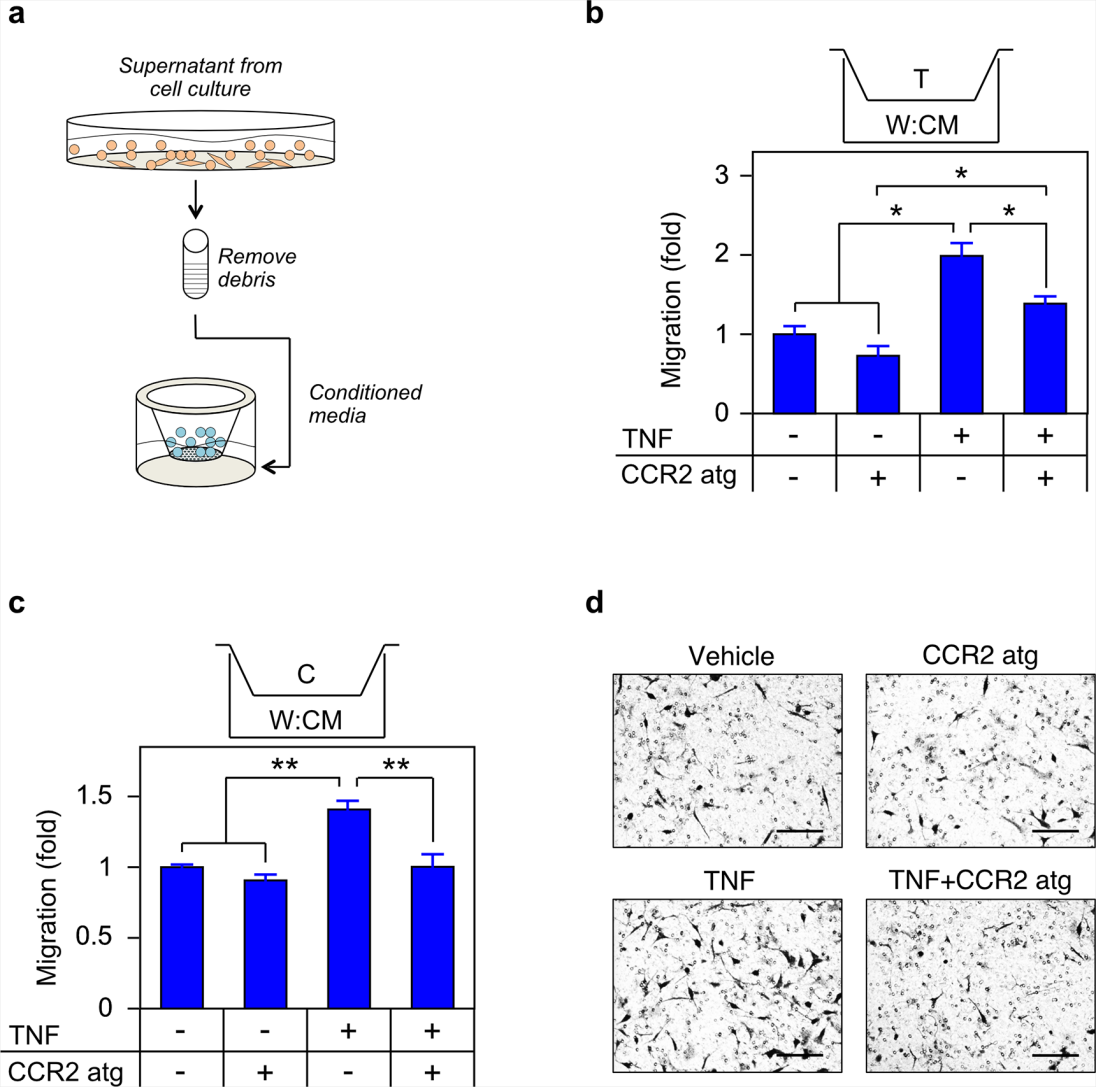
TNF induced CCL2 signaling is required for migration **a.** Schematic of migration assay. **b. c.** THP1 (b) or C4-2 (c) were seeded in the upper chamber and migration determined as in Materials and Methods (*n* = 3). CM was from cells grown for 72 h in media plus vehicle (−) or 100 ng/mL TNF (TNF, +). The CCR2 antagonist (CCR2 atg, +) was added to the CM, as indicated. **d.** Representative photomicrographs of migrated C4-2 cells from (c) Magnification, 460 ×; scale bar, 100 μm. Differences were assessed by one-way ANOVA followed by Tukey-Kramer HSD test. *p < 0.05, **p < 0.01.

**Figure 9:**
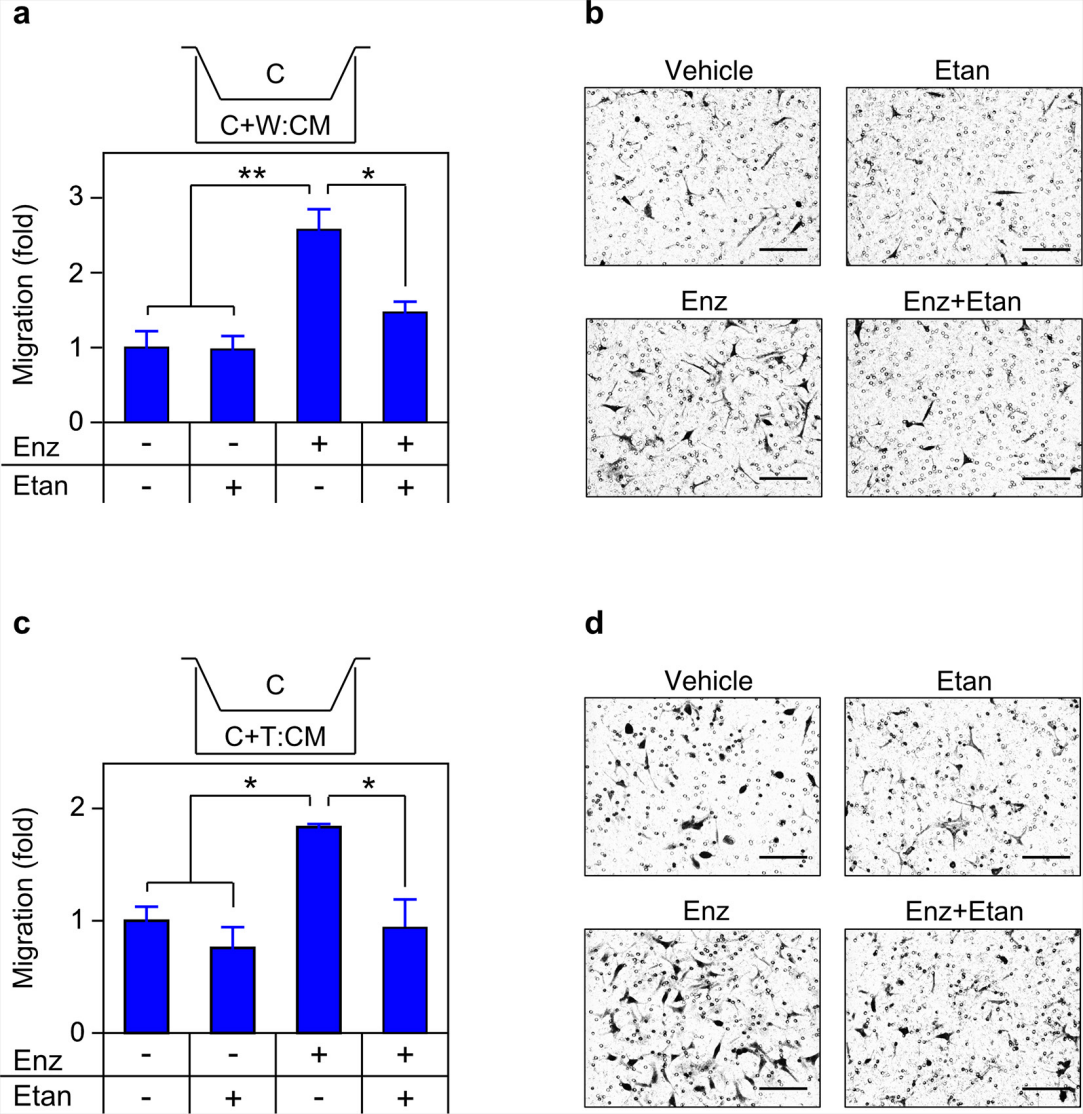
Secreted TNF is required for enzalutamide-induced migration **a. c.** Transwell migration assays were performed as in Figure 8, with C4-2 cells seeded in the upper chamber and migration determined as in Materials and Methods (*n* = 3). CM derived from mixed cultures of C4-2/WPMY-1 (a) or C4-2/THP1 (c) grown for 72 h in the presence of vehicle (−), 10 μM enzalutamide (Enz, +) or 1 μg/mL etanercept (Etan; +), as indicated. **b. d.** Representative photomicrographs of migrated C4-2 cells from (a) and (c) respectively. Magnification, 460x; scale bar, 100 μm. Differences were assessed by one-way ANOVA followed by Tukey-Kramer HSD test. *p < 0.05, **p < 0.01

**Figure 10:**
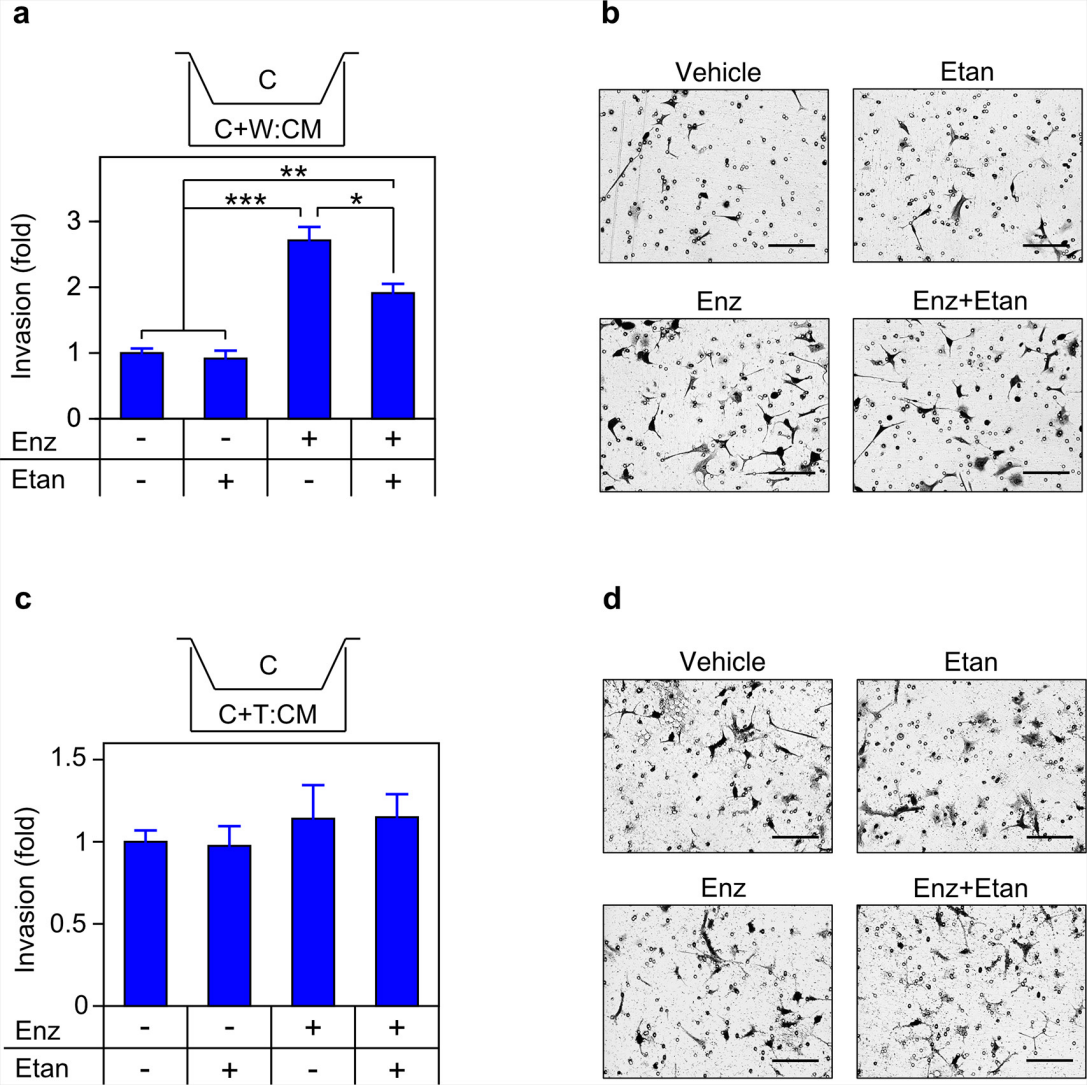
Secreted TNF is required for enzalutamide-induced invasion **a. c.** Transwell invasion assays were performed, essentially identical to Figure 9, except that transwell membranes were coated with Matrigel, (*n* = 3). **b. d.** Representative photomicrographs of cells that have invaded the Matrigel coated membrane, from (a) and (c) respectively. Magnification, 460 ×; scale bar, 100 μm. Statistical comparison by one-way ANOVA followed by Tukey-Kramer HSD test. *p < 0.05, **p < 0.01, ***p < 0.001

As noted, we have previously demonstrated that enzalutamide induces a metastatic phenotype in both cell culture and mouse models of CRPC [11]. To determine if TNF regulation of CCL2 is required for the metastatic phenotype, we performed three experiments. First, we demonstrated that TNF induces migration of tumor associated macrophage-like THP-1 and CRPC C4-2 cells in a cell culture model of the tumor microenvironment (Figure 8). Specifically, conditioned media (CM) from TNF-treated WPMY-1 cells, expected to contain biologically active concentrations of CCL2 (Figure 6g), was placed in the bottom of a transwell chamber (Figure 8a). Both THP-1 and C4-2 cells migrated towards the CM, and migration was reduced by a CCR2 inhibitor (Figure 8b–8d). Notably, neither C4-2 nor THP-1 cells migrated towards media plus recombinant TNF (Supplementary Figure S3). Also, TNF did not enhance C4-2 proliferation (Supplementary Figure S4), arguing that increased cell numbers do not account for increased migration. Second, paracrine CCL2, produced by enzalutamide treatment, induced migration of C4-2 (Figure 9). In these experiments, CM was from enzalutamide-treated C4-2/WPMY-1 (Figure 9a, 9b) or C4-2/THP-1 (Figure 9c, 9d) mixed cultures. Again, the enzalutamide-treated CM is expected to contain higher levels of CCL2 (relative to the vehicle-treated control), in this case due to enzalutamide induction of TNF (Figure 7). C4-2 migration towards the CM was inhibited when etanercept was present during CM production, thereby blocking TNF-mediated paracrine CCL2 secretion. Finally, we replicated the experiment in Figure 9, but measured invasion, i.e., migration through a Matrigel-coated membrane. C4-2 cells were induced to invade, in a TNF-dependent manner, by C4-2/WPMY-1 CM (Figure 10a, 10b) but not by C4-2/THP-1 CM (Figure 10c, 10d), consistent with the relatively higher level of TNF-dependent CCL2 production by WPMY-1 cultures (Figure 6g).

### TNF and CCL2 expression in prostate cancers

**Figure 11:**
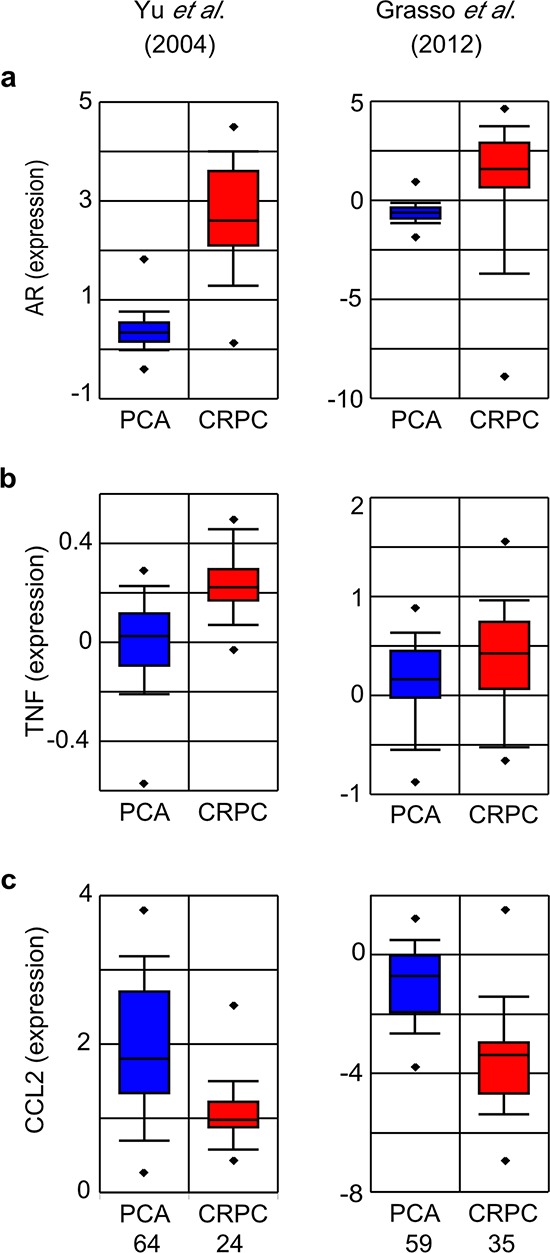
TNF, CCL2 and AR mRNA expression in metastatic CRPC Oncomine was used to generate box plots of AR **a.** TNF **b.** and CCL2 **c.** mRNA expression in human primary PCa (blue) and CRPC (red) from the indicated data sets. Sample size as indicated (bottom), with the exception that the Grass *et al*. data set contains only 34 (rather than 35) samples for the TNF probe. PCa and CRPC sample sets were compared by Student's unpaired *t*-test. *p* < 10^−5^, except **b.** Grasso *et al*., *p* = 0.02.

It is important to consider our findings in the context of human cancers. A key study [16] sampled PCa patients within three months of initiating ADT, when AR signaling is still likely suppressed (i.e., prior to the onset of CRPC), and found elevated serum TNF and CCL2 correlated with reduced survival. To gain additional insight into the correlation between PCa metastasis, CCL2 and TNF, we interrogated two sets of metastatic CRPC patient samples [33, 34] using Oncomine [35]. In these patients AR signaling is typically reactivated, and indeed *AR* mRNA expression levels are increased in both data sets (Figure 11a). *TNF* mRNA was significantly elevated in CRPC patient samples (Figure 11b; see also Supplementary Figure S5). In contrast, *CCL2* mRNA was significantly reduced in both data sets (Figure 11c), which may reflect induction of CCL2 at the primary tumor site only [48], rather than also at the metastatic tumor site (see Discussion). Moreover, a similar pattern (AR increased, CCL2 significantly decreased) is observed in metastases from patients who did not receive ADT (Supplementary Figure S6, Supplementary Table S1), supporting the idea that CCL2 is only required at the primary tumor site.

## DISCUSSION

Observations that TNF can transcriptionally regulate the *CCL2* gene [18], that NFκB is up-regulated in PCa [36–38], and that ADT induced metastasis is dependent on CCL2 [11–13], led us to test the hypothesis that TNF mediates the paradoxical induction of metastasis that occurs following ADT. The role of TNF in PCa has been uncertain [39]. Castration-induced regression (due to epithelial cell apoptosis) in normal rodent prostate requires TNF up-regulation [17] and down-regulation of the caspase-8 inhibitor c-FLIP [40], but in CRPC c-FLIP expression is increased [41], blocking the pro-apoptotic effects of TNF and inducing NFκB, which promotes cell survival [42]. The induction of metastases by ADT, even as the primary tumor regresses [11–13], suggests a stage-specific role for TNF in CRPC, similar to TGFΔ, which inhibits early cancers and promotes late stage cancer metastasis [43, 44]. First we demonstrated that in C4-2 CRPC tumor cells, various modes of androgen deprivation de-repress *TNF* mRNA expression, leading to secretion into the media (Figures 1–2). Enzalutamide-induced *CCL2* mRNA and protein is dependent on secreted TNF in these cells (Figure 3–4), since it is blocked by a soluble receptor. Figure 5 demonstrates that ADT-induced TNF signaling can enhance CCL2 secretion in a second CRPC-derived cell line (PTEN-CaP8), but not in the less advanced parental cell lines that gave rise to both C4-2 and PTEN-CaP8. This suggests the possibility that a common mechanism driving the evolution of CRPCs also selects for CCL2 expression in response to TNF signaling (likely among other pro-tumorigenic features). In support of this concept, Qin *et al*. [45] found that ADT of LNCaP cells enriches for a minority LNCaP cell population (PSA^−/lo^ cells) that displays a castration-resistant cancer phenotype, including the ability to grow in castrate hosts, and enhanced expression of cancer stem cell genes. A similar selection process may have been at work in the development of C4-2 and PTEN-CaP8 from the respective parental cell lines LNCaP and PTEN-P8 [24, 25, 31]. Thus, we speculate that the ADT-induced production of CCL2 via TNF signaling is a feature of a population resembling PSA^−/lo^ (progenitor) cells, and contributes to the aggressive tumor phenotype of this cancer population, which expands following ADT [45].

**Figure 12:**
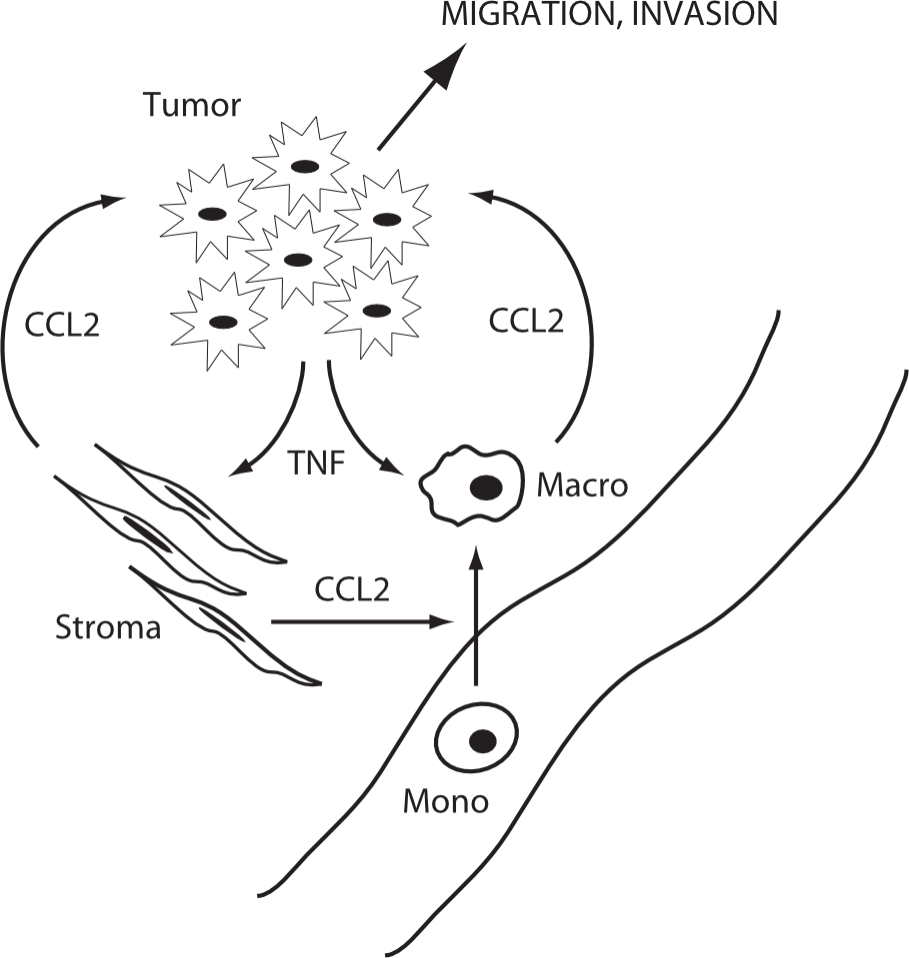
Schematic of microenvironment interactions Tumor cells produce TNF in response to ADT, which induces CCL2 in stromal cells and macrophages, and promotes tumor cell migration and invasion and monocyte migration and differentiation to macrophages.

Exogenously added TNF induced *CCL2* mRNA and CCL2 protein in macrophage- and myofibroblast-like cells, suggesting paracrine regulation of CCL2 production (Figure 6). We confirmed the paracrine mechanism by employing mixed cultures of tumor and microenvironment cells (Figure 7) and, analogously, by measuring *in vitro* metastatic phenotypes (migration and invasion) that depend on CCL2 signaling. Again, exogenous cytokine addition or conditioned media – both mimicking possible paracrine effects within the microenvironment - demonstrated a functional role for TNF in the paracrine loop controlling metastasis in response to enzalutamide (Figures 8–10). We therefore propose that tumor cells secrete TNF, which induces CCL2 production in two microenvironment cells (myofibroblasts and macrophages) promoting tumor migration, invasion and extravasation [46] and monocyte recruitment and differentiation into tumor associated macrophages [12, 14]. Figure 12 illustrates the paracrine interactions mediating ADT induced metastasis via the chemotactic activities of CCL2.

In our model system, enzalutamide induces TNF modestly and only in CRPC cells, but is sufficient to up-regulate *CCL2* mRNA expression and CCL2 protein secretion, particularly in the WPMY-1 myofibroblast cells. Previously, we had shown that castration of rodents induced *TNF* mRNA expression in stromal, but not the epithelial cells of the normal prostate [17]. The observations reported here, that androgen deprivation induces *TNF* mRNA expression in an epithelial tumor cell line, but not the myofibroblast stromal cell line, may reflect differences inherent in tumor and tumor-associated cells as a result of the process of tumorigenesis. As expected, there is a correlation between the level of TNF secretion and the extent of invasion, which is a more stringent criteria of the metastatic phenotype (Figure 10). Although cell culture models facilitated our identification of key paracrine interactions in the cancer microenvironment, there are limitations in extrapolation to *in vivo* situations. In particular, our culture conditions may not reproduce the proportions of cellular constituents or the effective concentration of the secreted ligands seen *in vivo*. However, while our data derives from the co-culture of a set of relevant cell lines, many aspects of this signaling paradigm have been confirmed in murine models. Specifically, our prior data demonstrate that conditional deletion or silencing of AR in macrophages and PCa xenografts induces CCL2, macrophage infiltration of xenografts and metastasis [12]. Previously we showed that enzalutamide induces CCL2 in TRAMP-C1 xenografts, and that castration similarly induces CCL2 in normal mouse prostate [12]. Moreover, enzalutamide-enhanced metastasis has been observed in other CRPC models [47]. Future studies in mouse models are needed to confirm the microenvironment source(s) of TNF and verify a functional role for TNF in ADT induced metastasis *in vivo*.

Finally, a variety of data from PCa patients support our hypothesis. For example, we found CCL2 expression in human PCa samples correlated with PSA levels, the number of infiltrating macrophages and poor prognosis [12]. In a small patient study, anti-androgens enhance the growth of metastases, indicating that ADT-induced metastasis occurs in human PCa [13]. The strongest human data in support of a TNF-CCL2 paracrine loop comes from PCa patients assayed within three months of initiation of ADT [16]. At this stage of treatment, androgen signaling will be fully repressed (patient serum PSA levels low). In this cohort, patients with increased TNF and CCL2 fared worse, consistent with therapy-induced TNF/CCL2 enhancing metastasis even though ADT inhibited the growth of primary tumors. As mentioned above, this implies a divergence between AR signaling effects on proliferation and the metastatic phenotype.

While immunohistochemical staining of primary (localized) PCa tumors showed CCL2 levels correlated with Gleason score and tumor stage [48], analysis of metastasized CRPC sample data sets in Oncomine reveals reduced CCL2 (Figure 11). Specifically, in CRPC patients (where AR signaling is likely reactivated, and patient serum PSA levels are rising) *CCL2* mRNA in the metastases was clearly reduced. Similarly, metastases in patients who were not given ADT also have decreased *CCL2* mRNA (Supplementary Figure S6). Since the tumor cells have already metastasized and escaped the primary tumor microenvironment, CCL2 may no longer be necessary. That is, once metastatic seeding has occurred, the role of CCL2 in monocyte differentiation and tumor cell migration is diminished. Moreover, the stromal tumor microenvironment that was present in the primary tumor is unlikely to be replicated at the site of the metastatic lesion(s), which lack the cellular interactions that we believe generate CCL2. Furthermore, TNF is significantly elevated in the CRPC metastatic samples (Figure 11), but not consistently elevated in non-CRPC samples. The comparisons of non-CRPC patient samples include primary tumors, which have low versus high Gleason scores (Supplementary Figure S7), as well as primary versus metastasis from patients who did not undergo ADT (Supplementary Figure S6). Taken together, these latter datasets suggest that TNF induction is only consistently seen in response to ADT therapy, rather than as a consequence of tumor progression.

We therefore propose that CCL2 primarily acts via a therapy-induced paracrine loop, such as the one we document in cell culture models, at the site of the primary tumor but not at the site(s) of metastases. Another way to view this is that the process of metastasis interrupts the paracrine loop (TNF®CCL2) by relocating tumor cells away from the original tumor microenvironment. This further suggests that therapies directed against CCL2 or TNF might be effective only during a narrow window of time; that is, only if administered in conjunction with the initial course of androgen deprivation therapy given to patients still possessing a primary tumor, prior to the onset of CRPC and metastases. Thus, our mechanistic observations may explain the recent failure of CCL2 directed monoclonal therapy in PCa [49] since these trials were performed using anti-CCL2 as a single agent and in post-ADT patients who had already developed metastatic CRPC.

## MATERIALS AND METHODS

### Reagents

The following reagents were used: DHT (Sigma, St. Louis, MO, USA), enzalutamide (Selleckchem, Houston, TX, USA), etanercept (Amgen, Thousand Oaks, CA, USA), R1881 (PerkinElmer, Waltham, MA, USA), CCR2 antagonist BMS-741672 (Torcis Bioscience, Bristol, UK), and TNF (BioLegend, San Diego, CA, USA).

### Cell culture

The C4-2 cell line was from Dr. Jer-Tsong Hsieh, UT Southwestern Medical Center, Dallas, TX. LNCaP, THP1, WPMY-1, PTEN-P8 and PTEN-CaP8 cell lines were from the American Type Culture Collection (Rockwell, MD, USA). C4-2, WPMY-1, THP1 and LNCaP, were cultured in RPMI-1640 media supplemented with 10% FCS and 1% penicillin/streptomycin. For THP1, the media also contained 10 mM HEPES and 2 mM glutamine; LNCaP culture media also contained 1 mM sodium pyruvate. PTEN-P8 and PTEN-CaP8 were cultured in DMEM supplemented with 10% FCS and 1% penicillin/streptomycin.

### Enzyme-linked immunosorbent assays (ELISA)

Cells were cultured at a density of 1 × 10^5^/ml and supplemented with enzalutamide, DHT or etanercept as indicated. For TNF treatment, cells were cultured at 4 × 10^4^/ml. Cells were seeded at 5 × 10^4^/ml for C4-2/WPMY-1 co-cultures and 1 × 10^5^/ml for C4-2/THP1 co-cultures. TNF and CCL2 were quantitated with ELISA kits (eBioscience, San Diego, CA, USA), per the manufacturer's instructions.

### Metastasis assays

Two *in vitro* transwell assays (migration, invasion) were performed to assess metastatic phenotypes. For migration assays, cells were serum starved for 24 h, trypsinized if required, washed and resuspended in serum-free media. Approximately 1 × 10^5^ cells were placed in the upper chamber of a 24-transwell plate (Corning Inc., Tewksbury, MA, USA). Matrigel coated chambers (Biocoat, Corning, NY, USA) was added to wells used for invasion assays. Conditioned medium was prepared by diluting culture supernatants with an equal amount of fresh media plus 5% FCS. For C4-2 cells, after 22 h incubation, chambers were fixed and stained with 1% toluidine blue for manual counting of photomicrographs under the microscope (Life Technologies, Carlsbad, CA, USA). For THP-1 cells, after 16 h incubation, media was collected and cells counted by Coulter counter. Counts were normalized to vehicle control.

### RNA isolation and quantitative RT-PCR

Cells were cultured at the same densities used for the ELISA assays. Total RNA was extracted and reverse transcribed using pdN15 with Superscript III reverse transcription kit (Invitrogen, Waltham, MA, USA) to generate single-strand cDNA. PCR was performed using SYBR^®^ Green qPCR Master Mix (Molecular Probes, Eugene, OR, USA) in a StepOnePlus instrument (Applied Biosystems, Waltham, MA, USA). PCR primers, spanning at least one intron, were: TNF-forward: 5′-CCTCTCTCTAATCAGCCCTCTG-3′; TNF-reverse: 5′-GAGGACCTGGGAGTA-GATGAG-3′; CCL2-forward: 5′-CAGCCAGATGCAATCAATG-CC-3′; CCL2-reverse: 5′-TGGAATCCTGAACCCACTTCT-3′; β-actin-forward: 5′-CATGTAC-GTTGCTATCCAGGC-3′ and β-actin reverse: 5′-CTCCTTAATGTCACGCACGAT-3′. Standard curves were generated using linearized plasmids encoding the corresponding cDNAs. β-Actin was used to normalize target gene expression. At least three biological replicates were analyzed (unless otherwise indicated), and at least three trials were conducted for each sample.

### Cell proliferation assay

C4-2 cells at 50%-75% confluence were trypsinized and seeded into 96-well plates at 4.5 × 10^3^ cells/well. Cells were cultured in complete medium supplemented with 100 ng/ml TNF for 72 h. Viability was measured by WST-1 assay, as per the manufacturer (Roche Applied Science, Indianapolis, IN, USA).

### Microarray database analysis

Plots of gene expression levels were obtained from Oncomine (Life Technologies, http://www.oncomine.org). For statistical analysis individual data values were extracted from the Oncomine database. Homology regions of microarray probes were determined using UCSC genome browser (http://genome.ucsc.edu).

### Statistics

Data are presented as the mean ± SEM. Statistical analyses were conducted using JMP-Pro10 (SAS, Cary, NC, USA). Differences between two means was assessed by Student's unpaired *t*-test and differences among multiple means by one-way ANOVA followed by Tukey-Kramer HSD test.

## SUPPLEMENTARY FIGURES AND TABLE


