# Supplementary material for: TNF signaling mediates an enzalutamide-induced metastatic phenotype of prostate cancer and microenvironment cell co-cultures
Source: Oncotarget. 2015 Jul 30;6(28):25726–40. doi: 10.18632/oncotarget.4535 (PMC4694862; doi:10.18632/oncotarget.4535)
Supplement: Supplementary file 1 [file oncotarget-06-25726-s001.pdf]

SUPPLEMENTARY FIGURES AND TABLE

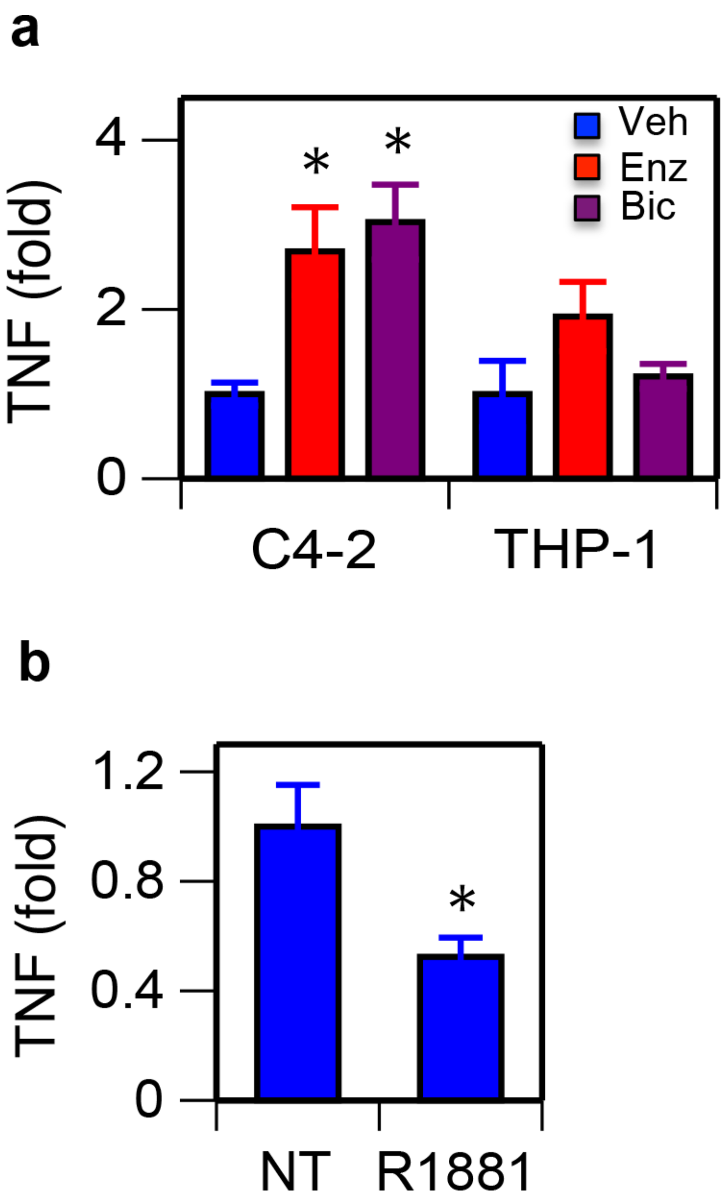

Supplementary Figure S1: ADT induces TNF secretion of CRPC following anti-androgen and synthetic androgen treatment. a. THP1 or C4-2 were treated with vehicle control (NT), 10 µM enzalutamide (Enz) or 10 µM bicalutamide (Bic), and incubated with 10 nM DHT for 72 h. **b.** C4-2 was treated ± 1 nM R1881. TNF was assayed by ELISA ($n \geq 3$). Student's unpaired *t*-test was used to assess differences between controls and treated groups. *$p < 0.05$.

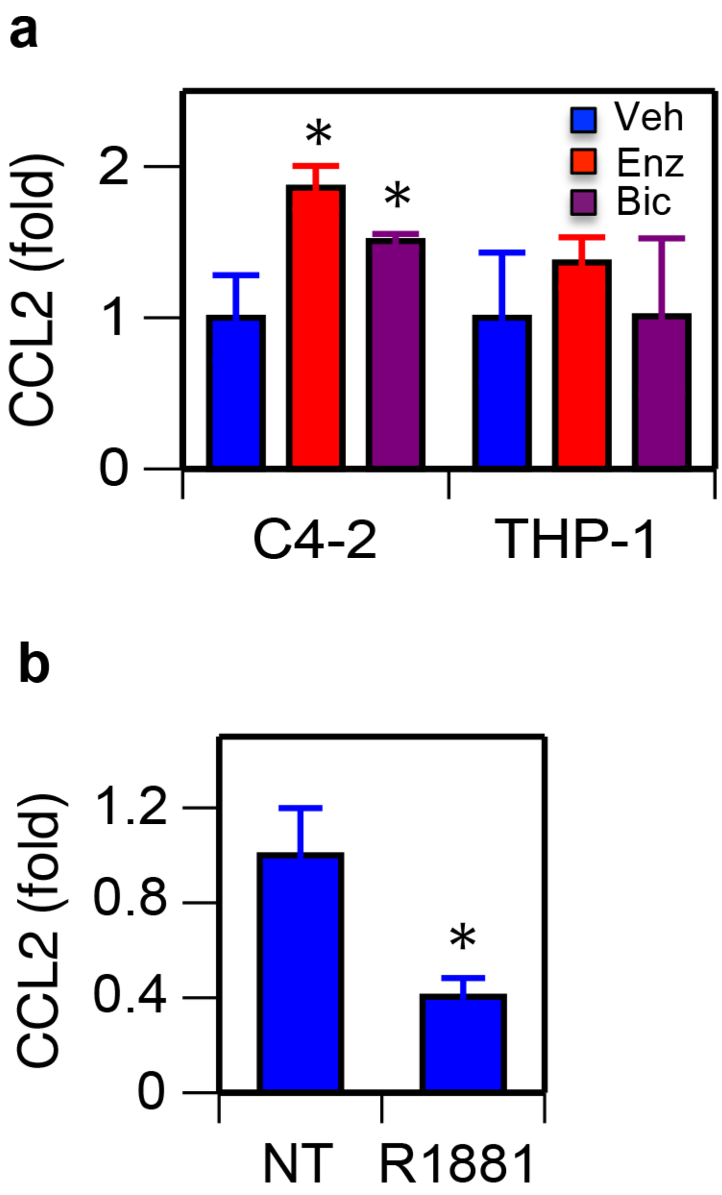

Supplementary Figure S2: ADT induces CCL2 secretion of CRPC following anti-androgen and synthetic androgen treatment. a. THP1 or C4-2 were treated with vehicle control (NT), 10 µM enzalutamide (Enz) or 10 µM bicalutamide (Bic), and incubated with 10 nM DHT for 72 h. **b.** C4-2 was treated ± 1 nM R1881. CCL2 was assayed by ELISA ($n \geq 3$). Student's unpaired *t*-test was used to assess differences between controls and treated groups. *$p < 0.05$.

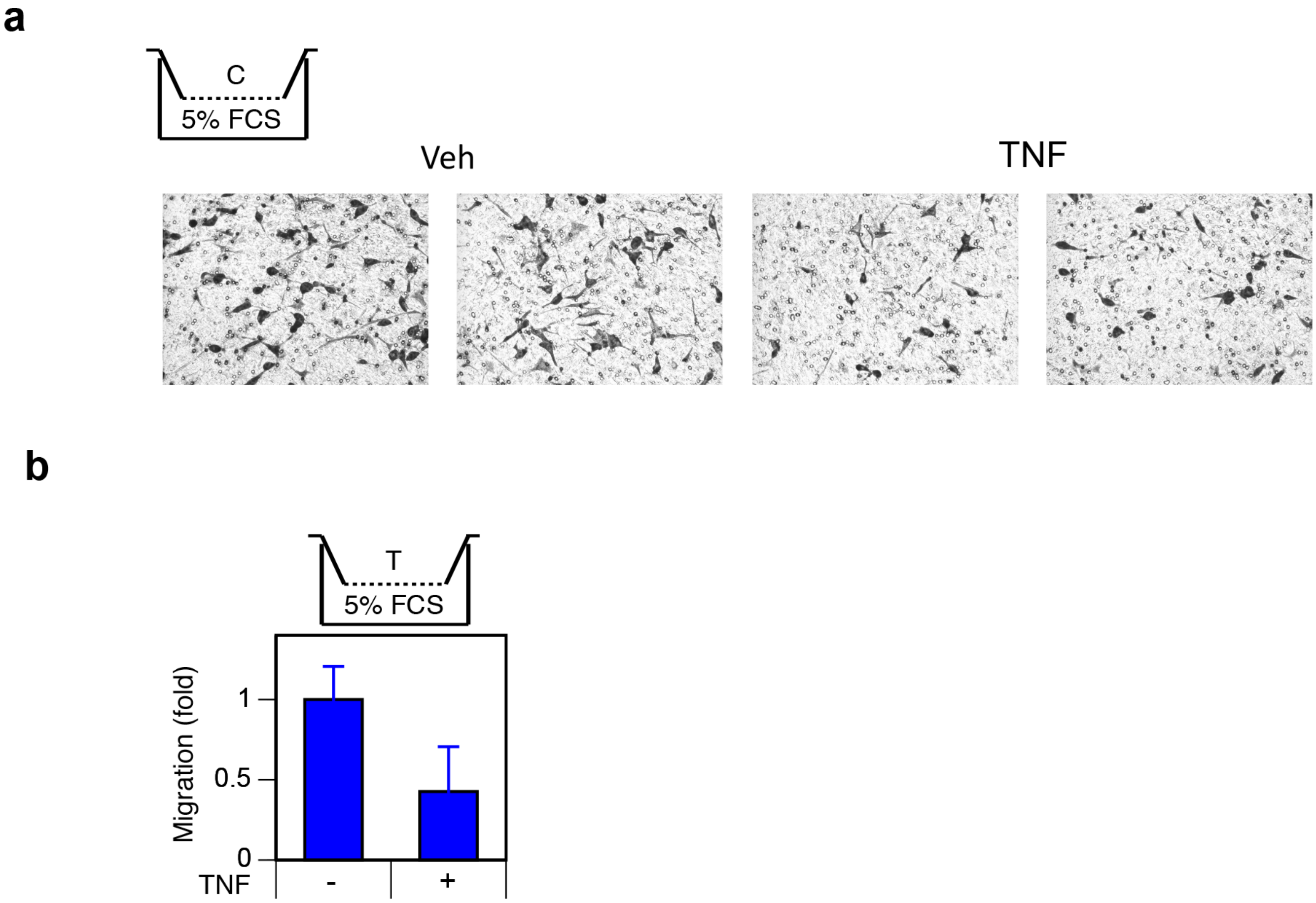

Supplementary Figure S3: Exogenously added TNF does not affect CRPC or macrophage-like cell migration. Transwell migration assays were performed as described in the Materials and Methods, with the upper chamber containing C4-2 (C) **a.** or THP1 (T) **b.** as indicated. The lower chamber contained media plus 5% FCS, ± 100 ng/ml TNF. Magnification = × 460, scale bar = 100 µm (*n* = 2). In (a) representative microphotographs were taken of migrated cells in the vehicle control and TNF cultures (cell counting revealed that there is no difference between vehicle and TNF). In (b) migrated cells were counted as described in the Figure 8, and are plotted as fold-change following normalization to the vehicle control (–) cultures.

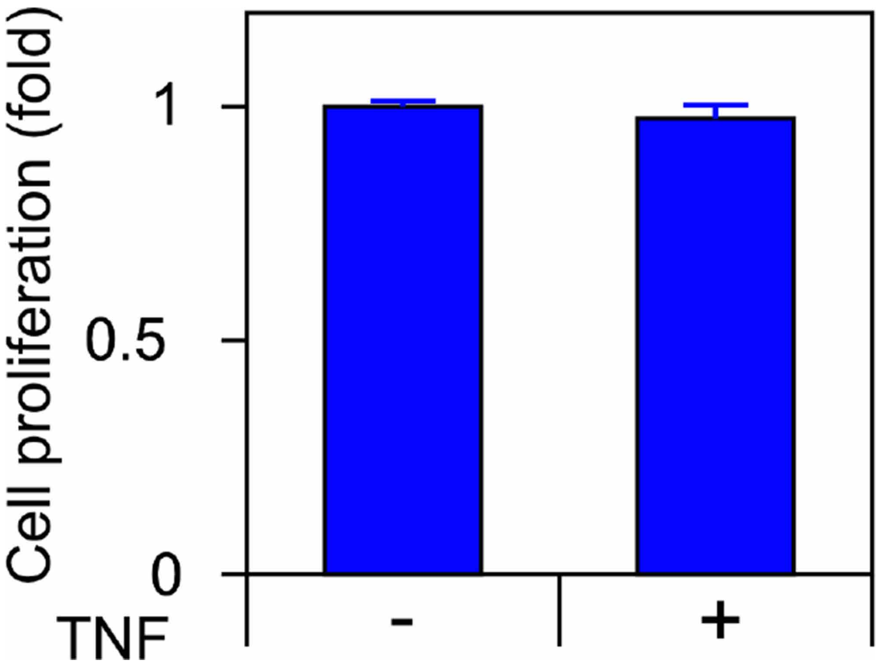

Supplementary Figure S4: TNF has no effect on the proliferation of CRPC cells. C4-2 cells were treated ± 100 ng/ml TNF and then incubated in the presence of WST-1 72 h later. Reduction of this tetrazolium dye was measured as described in the Materials and Methods to quantitate cell proliferation (*n* = 3).

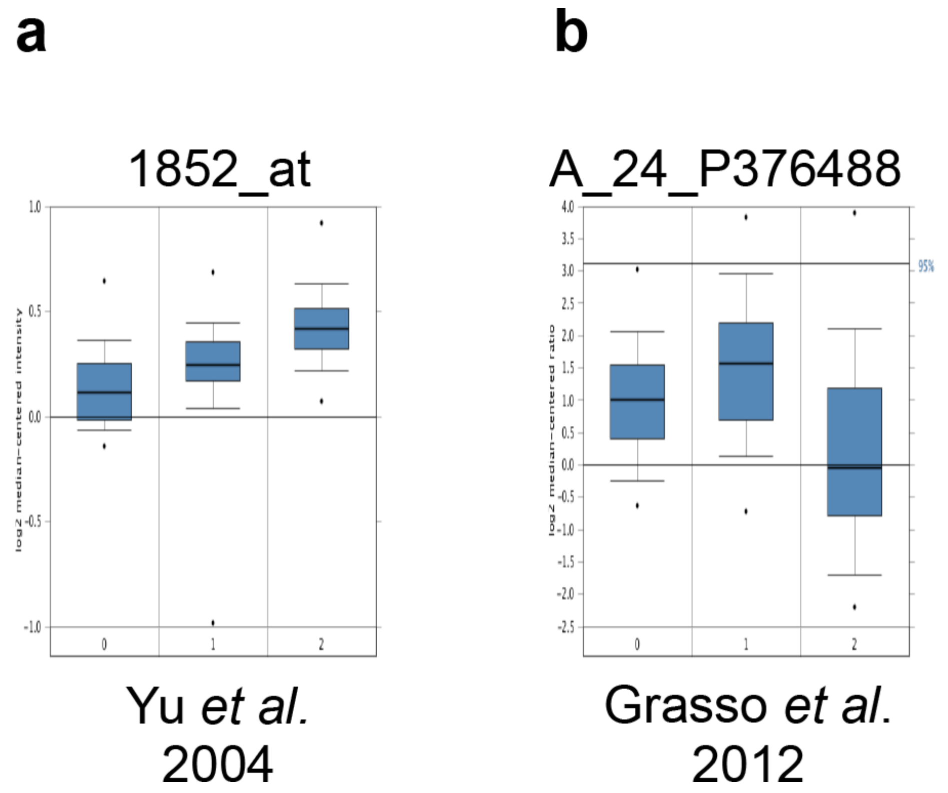

Supplementary Figure S5: Additional oncomine analyses of TNF expression in metastatic CRPC. Box-plots of TNF mRNA levels in human normal or benign hyperplastic prostate tissue (left-most boxes in each plot), primary prostate cancer (middle boxes) and castration resistant prostate cancer (right-most boxes) were extracted by Oncomine analysis from two published data sets: **a.** Yu *et al*. 2004 and **b.** Grasso *et al*. 2012. These data are both from 3' UTR probes while all the TNF data in Figure 9 is from coding region probes. There is substantial variability in 3' UTR sequences of mRNAs, and thus 3'UTR probes may not be reliable for predicting mRNA levels [see Wang & Seed (2003) Selection of oligonucleotide probes for protein coding sequences *Bioinformatics* 19:796]. In the case of Yu *et al*. 2004, the 3'UTR probe results are in agreement with the coding region probe in Figure 9, while the 3'UTR probe from Grasso *et al*. 2010 is not in agreement.

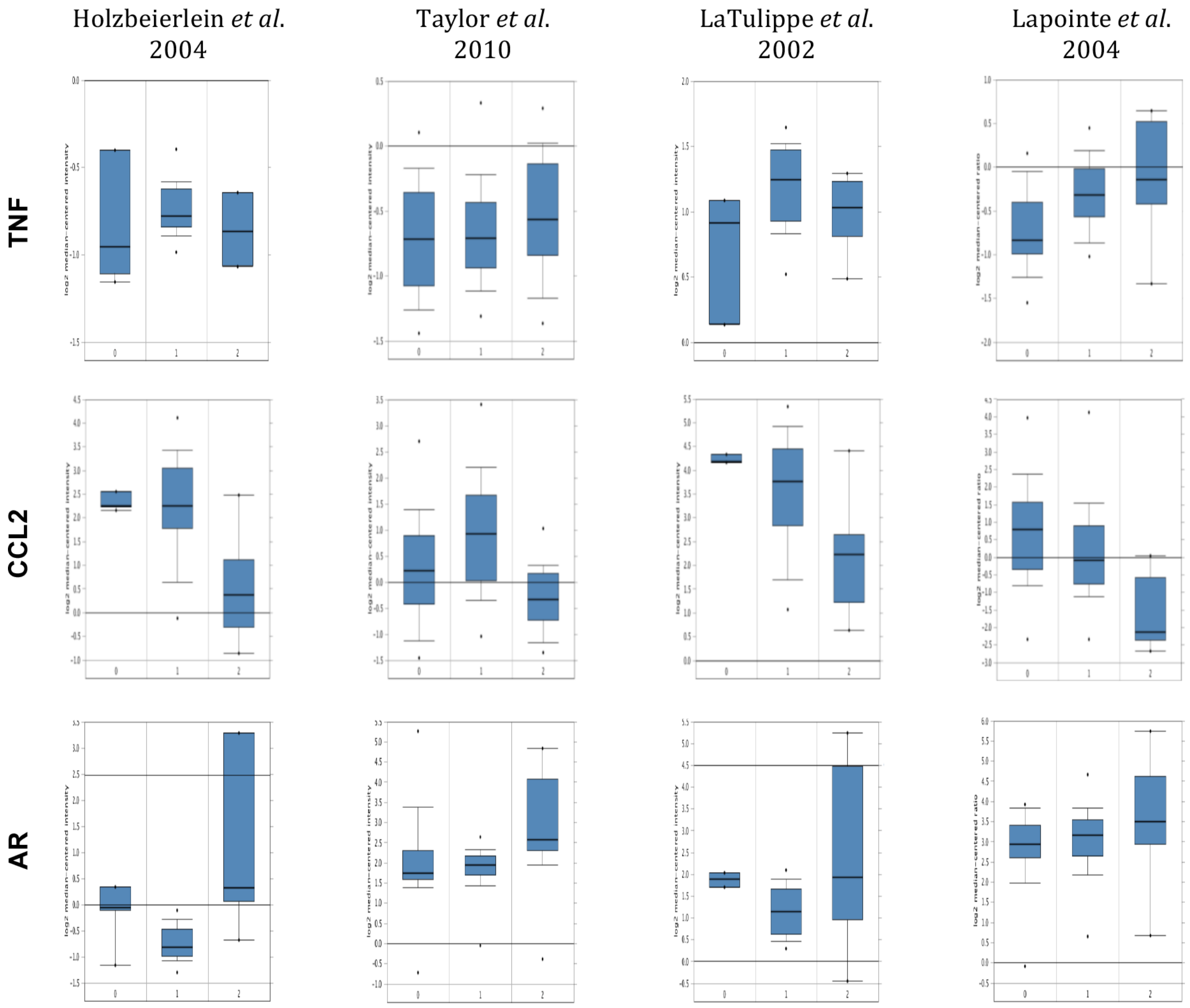

Supplementary Figure S6: Oncomine analysis of TNF, CCL2 and AR expression in metastatic PCa, not previously treated with ADT. Box plots of TNF, CCL2 and AR mRNA levels in human normal or benign prostate tissue (left-most boxes in each plot), primary prostate cancer (middle boxes) and castration resistant prostate cancer (right-most boxes) were extracted by Oncomine analysis. Source of the data sets is indicated on the top of the plots. Details about probes, number of samples and *p* values are in Table S1.

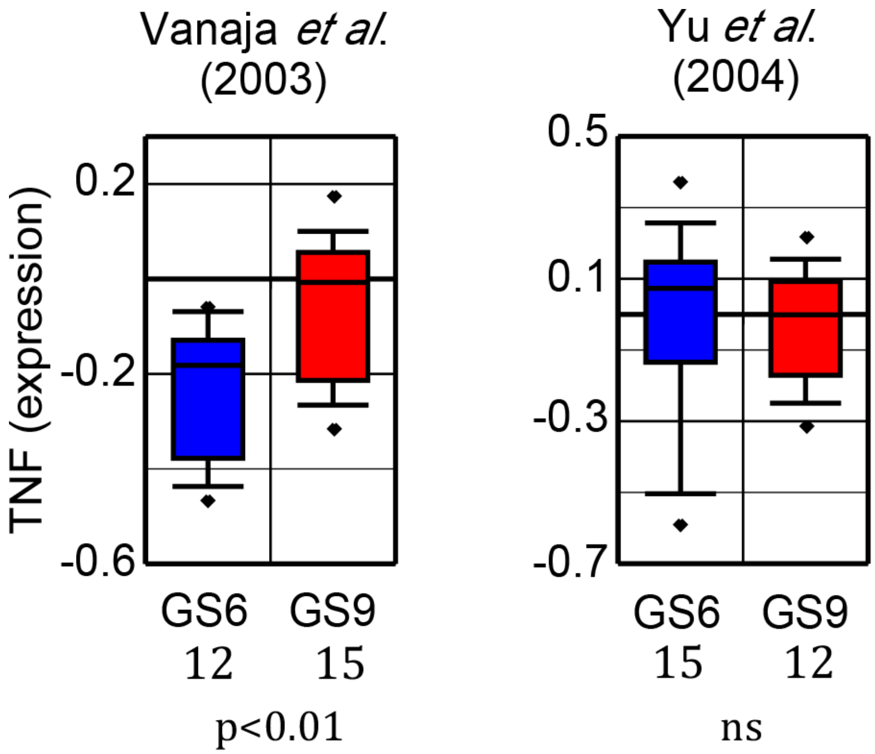

Supplementary Figure S7: Oncomine analysis of TNF expression in human primary prostate cancers stratified by Gleason score (GS). Box-plots of TNF mRNA levels in GS6 (blue) or GS9 (red) were extracted by Oncomine analysis from two published data sets: **a.** Vanaja et al. and **b.** Yu *et al*. 2004. Sample size as indicated under the GS. Student's unpaired *t*-test was used to assess differences. *P* values as indicated.

Supplementary Table S1: Sample size and probe IDs for four non-hormone-treated metastatic PCa microarray studies

Gene	Oncomine study	Holzbeierlein		Taylor		LaTulippe		Lapointe	
**TNF**	Sample Size	PCa	Met	PCa	Met	PCa	Met	PCa	Met
		13	6	131	19	23	9	57	9
	Probe ID	*1852_at*		*558*		*259_s_at*		*IMAGE:446927*	
**CCL2**	Sample Size	PCa	Met	PCa	Met	PCa	Met	PCa	Met
		40	9	131	19	23	9	62	8
	Probe ID	*875_g_at*		*7493*		*875_g_at*		*IMAGE:768561*	
**AR**	Sample Size	PCa	Met	PCa	Met	PCa	Met	PCa	Met
		36	7	131	19	23	9	61	9
	Probe ID	*1578_g_at*		*31*		*1578_g_at*		*IMAGE:1203148*	
